# Intranasal Delivery of Genistein-Loaded Nanoparticles as a Potential Preventive System against Neurodegenerative Disorders

**DOI:** 10.3390/pharmaceutics11010008

**Published:** 2018-12-29

**Authors:** Giovanna Rassu, Elena Piera Porcu, Silvia Fancello, Antonella Obinu, Nina Senes, Grazia Galleri, Rossana Migheli, Elisabetta Gavini, Paolo Giunchedi

**Affiliations:** 1 Department of Chemistry and Pharmacy, University of Sassari, via Muroni 23/a, 07100 Sassari, Italy; grassu@uniss.it (G.R.); elena.piera1988@gmail.com (E.P.P.); nsenes@uniss.it (N.S.); pgiunc@uniss.it (P.G.); 2 Department of Clinical and Experimental Medicine, University of Sassari, viale San Pietro 43/b, 07100 Sassari, Italy; sfancello@uniss.it (S.F.); galleri@uniss.it (G.G.); rmigheli@uniss.it (R.M.); 3 Experimental Medicine, Department of Clinical-Surgical, Diagnostic and Paediatric Sciences, University of Pavia, 27100 Pavia, Italy; antonella.obinu01@universitadipavia.it

**Keywords:** chitosan, genistein, sodium hexametaphosphate, nose-to-brain, intranasal delivery, neurodegenerative disease, nanoparticles

## Abstract

Genistein has been reported to have antioxidant and neuroprotective activity. Despite encouraging in vitro and in vivo results, several disadvantages such as poor water solubility, rapid metabolism, and low oral bioavailability limit the clinical application of genistein. The aim of this study was to design and characterize genistein-loaded chitosan nanoparticles for intranasal drug delivery, prepared by the ionic gelation technique by using sodium hexametaphosphate. Nanoparticles were characterized in vitro and their cytotoxicity was tested on PC12 cells. Genistein-loaded nanoparticles were prepared, and sodium hexametaphosphate was used as a valid alternative to well-known cross-linkers. Nanoparticle characteristics as well as their physical stability were affected by formulation composition and manufacturing. Small (mean diameters of 200–300 nm) and homogeneous nanoparticles were obtained and were able to improve genistein penetration through the nasal mucosa as compared to pure genistein. Nanoparticle dispersions showed a pH consistent with the nasal fluid and preserved PC12 cell vitality.

## 1. Introduction

Neurodegenerative diseases (NDDs) such as Alzheimer’s and Parkinson’s diseases are a major cause of disability and premature death among aged individuals worldwide [1]. These disorders are characterized by slowly progressive deterioration of structure or function of neurons [2,3]. The etiology of NDDs has not yet been fully clarified; however, they seem to be a consequence of multiple factors leading to neuronal degeneration. A combination of several factors such as increasing age, mitochondrial dysfunction, oxidative stress, and/or environmental factors (i.e., exposure to metal toxicity, pesticides, electromagnetic fields) may be implicated in causing neurodegenerative syndromes [4,5]. Particularly, the oxidative stress plays a central role in a common pathophysiology of NDDs. Oxidative stress is induced by a disequilibrium redox state, due to the excessive production of reactive oxygen species or the dysfunction of the antioxidant system [3]. The brain is highly vulnerable to the effects of free radicals because of its high oxygen consumption, the abundant presence of lipid cells susceptible to peroxidation, and low regenerative capacity [6,7]. Antioxidant therapy has been suggested for the prevention and treatment of NDDs [8,9]. In the last decades, there has been a growing interest in the role of antioxidants in experimental and clinical medicine. Indeed, the potential beneficial role of these compounds in neurologic disease has been supported by several in vitro and in vivo studies. Genistein (GEN), an isoflavonoid phytoestrogen, has been known for its potential pharmacological properties [10,11] especially in the brain protection [12,13]. Due to its chemical structure, GEN is similar to endogenous estrogen and it exhibits effects by binding to the cell membrane and intracellular estrogen receptors [14]. Several studies have revealed that GEN can prevent tumors, cardiovascular disease, menopausal symptoms and improve the immune function of organisms [15,16,17,18]. Furthermore, the neuroprotective effect of GEN has been widely evaluated. In particular, the role of the isoflavone in the prevention and treatment of NDDs by reducing the deposition of Aβ, inflammatory damage, and calcium levels has been confirmed [19,20,21]. Despite encouraging in vitro and in vivo results, several drawbacks such as poor water solubility, rapid metabolism and excretion, and low oral bioavailability limit the clinical applications of GEN [22,23]. For these reasons, new efficient delivery systems need to be investigated, particularly taking into account that the drugs suggested for NDDs must be capable of crossing the blood–brain barrier (BBB) [24]. The intranasal route has been proven to be a non-invasive and efficient strategy. The nasal administration route shows several advantages such as self-administration, low cost, and better compliance for the patient. Indeed, drugs or particles could reach the brain directly via the olfactory epithelium and/or via the trigeminal nerves [25,26,27]. In vivo experiments demonstrated that nano-sized drug carriers could enhance nose-to-brain delivery of drugs compared to drug solutions [28]. Although the nose-to-brain pathway proved to be useful for a variety of active compounds, the amount of drug accesses the brain is reported to be low, normally less than 0.1% [29], because of the site of administration (mucociliar clearance, drug degradation) [30,31]. Our recent work demonstrated that nanosized carriers are able to improve GEN internalization in PC12 cells, reducing ROS and the amount of apoptotic cells generated by H_2_O_2_ treatment, strengthening the neuroprotective activity of GEN [32]. Among different strategies to overcome the nasal mucosal barrier, polymeric nanoparticles have attracted great attention because they can efficiently deliver a wide range of therapeutic compounds; particularly, the employment of mucoadhesive polymers can increase nasal residence and the possibility of controlled and prolonged effects on drug release and intracellular uptake [33,34]. Among these polymers, chitosan shows promising results as nasal excipient due to its intrinsic properties including low toxicity, excellent biocompatibility, muco-adhesiveness, and the ability to transiently open the tight junctions between cells [35,36]. Different chitosan formulations have been proposed for the nose-to-brain delivery of drugs such as solutions, microspheres, microemulsion and gels [37,38,39,40,41]. Ionic gelation is the most common technique employed for the preparation of chitosan nanoparticles for intranasal route. This method entails the ionic interactions between the positively charged primary amino groups of chitosan and the multivalent counter ions, such as sodium tripolyphosphate, which is the most used cross-linking agent [42]. In order to extend the possible cross-linkers available to prepare chitosan nanoparticles, sodium hexametaphosphate was used in this work as alternative cross-linker to, for the first time, develop chitosan nanoparticles for nose-to-brain delivery of GEN. The six negative charges of sodium hexametaphosphate in the neutral and slightly basic medium offer more binding sites readily available for interaction compared to tripolyphosphate; therefore, due to the increased availability of the binding sites, a stronger ionic complexation with chitosan cationic charges is estimated [43]. This material is widely used in food industries due to its non-toxicity [44]. The formulations were characterized and ex vivo tested by using nasal mucosa. Experiments on PC12 cells as neuronal model cells were carried out in order to evaluate formulations cytotoxicity.

## 2. Materials and Methods

### 2.1. Materials

Chitosan (CS) (Chitoclear^®^ 1360, MW 35 kDa, 96% deacetylated) was supplied from Primex Ltd. (Siglufjordur, Iceland). Genistein (>98%) (GEN) was purchased from Farmalabor (Milan, Italy). Sodium hexametaphosphate (SHMP) was procured from VWR International (Poole, England). Acetic acid (glacial), acetonitrile, and methanol were of chromatographic grade and were purchased from Merck (Darmstadt, Germany). Dulbecco’s modified Eagle’s medium (DMEM/F12, HEPES, no phenol red), horse serum (HS), fetal bovine serum (FBS), and streptomycin/penicillin were acquired from Life Technologies Italia (Monza, Italy). Phosphate-buffered saline (NaCl 0.138 M; KCl 0.0027 M; pH 7.4, at 25 °C) solution, trypan blue (0.4%), and 3-(4,5-dimethyl-thiazol-2-yl)-2,5,diphenyltetrazoliumbromide (MTT, 97.5%) were purchased from Sigma-Aldrich (Milan, Italy).

### 2.2. Purification of Chitosan

The purification of CS was carried out according with the procedure based in the work of Nasti and co-workers [45]. Two grams of CS were dissolved in 160 mL of 2% *v*/*v* acetic acid solution in bidistilled water. Complete dissolution was achieved after 4 h of stirring. The solution was then boiled for 15 min in order to denature and precipitate any proteic contaminants. Subsequently, the mixture was centrifuged (Centrifuge 5702R, Eppendorf AG, Hamburg, Germany) for 10 min at 3000× *g*, and then the supernatant was removed and filtered through 0.8-μm pore size nitrocellulose membrane filter. The pH of the filtrated was then corrected to 9 with 1 N sodium hydroxide, in order to precipitate CS from the aqueous phase. After centrifugation, the precipitate was redispersed and again sedimented via centrifugation twice, always using bidistilled water at pH 9 as a dispersing medium. After storage at −80 °C, each sample was freeze-dried. In order to achieve appropriates freeze-dried powders to subsequent procedures, the samples were milled in a sealed milling system. The dried samples were used for the preparation of nanoparticles.

### 2.3. Preparation of Chitosan Nanoparticles

Chitosan particles were prepared by using ionotropic gelation technique [43,46] with some modifications.

A formulative study was carried out by changing parameters to obtain size of nanoparticles useful for nasal administration. CS concentration (0.5, 0.8, 1 and 2 mg/mL), CS:SHMP mass ratio (2:1, 2.5:1, 3:1, 4:1; 5:1) (Table 1), and the amount of GEN (0.5, 1.0, 2.0 mg) and organic solvent (acetone or 70° ethanol *v*/*v*) (Table 2) were the parameters considered. Briefly, CS was dissolved in aqueous acetic acid solution (1% *v*/*v*) and stirred for about 30 min in order to obtain the homogeneous solution. The pH was adjusted to 4.6 using 1 M NaOH. SHMP was dissolved in deionised water and added to CS solution at room temperature. For the preparation of GEN-loaded nanoparticles (Table 2), GEN was separately dissolved in two different organic solvents, acetone, or 70% (*v*/*v*) ethanol, and then added dropwise to chitosan solution with continuous stirring prior to addition of SHMP solution. The final solution was sonicated for 5 min at 35 Hz using an ultrasonic bath (Sonorex super RK 52 H, Bandelin electronic, Berlin, Germany). The SHMP solution, used as cross-linker, was added dropwise in the chitosan solution under continuous stirring (100 g) at room temperature and further stirred for 30 min. Nanoparticles were collected and washed twice with distilled water by using Amicon^®^ Ultra-15 (MWCO 30 kDa, Millipore, Merck KGaA, Darmstadt, Germany) at 3000× *g*, for 30 min at 4 °C. In the case of nanoparticles containing acetone, the concentration was carried out after the evaporation of the solvent under stirring. Blank CS nanoparticles were prepared by adding organic solvent without dissolving GEN and named with the codes B-NPAc and B-NPEt (Table 2).

### 2.4. Analysis of Particle Size and Polydispersity

Particle size was determined for all formulations. Blank and GEN-loaded nanoparticles were characterized for particle diameter and polydispersity index (PDI) by photo correlation spectroscopy (PCS) using a Coulter nanosizer N5 (Beckman-Coulter Inc., Miami, FL, USA). Before the analysis, 20 μL of nanoparticle dispersions was diluted with 3 mL distilled water in order to obtain the concentration required by the equipment (range 5 × 10^4^–1 × 10^6^ counts/s).

### 2.5. Stability Studies

The physical stability of blank nanoparticles and GEN-loaded nanoparticles (NPAc2 and NPEt1) was determined to investigate if significant changes in particle size and polydispersity index (PDI) occur during storage; the physical stability of formulations was evaluated to find the time useful for performing further tests and deduce at which conditions of temperatures the formulations were physically more stable. After preparation, the samples were stored at room temperature (25 °C) and 4 °C and dimensional properties were evaluated after 1, 7, and 14 days.

### 2.6. Determination of the Entrapment Efficiency

The entrapment efficiency (EE) was calculated by indirect method, evaluating the amount of unloaded GEN. The nanoparticles suspension prepared with acetone was kept under stirring for 12 h in order to allow the evaporation of the solvent and the precipitation of unloaded GEN. The employment of Amicon^®^ (MWCO 30 kDa, Millipore, Merck KGaA, Darmstadt, Germany) was avoided because the use of acetone is not recommended with the filter device. The suspensions were centrifuged at 3000× *g* for 20 min and the precipitate was resuspended with methanol (10 mL), appropriately diluted and filtered in order to quantify unloaded GEN.

With the formulation prepared with ethanol, the unloaded GEN was separated using filtration/centrifugation with Amicon^®^ Ultra-15 centrifugal filter devices (MWCO 30 kDa, Millipore, Merck KGaA, Darmstadt, Germany) at 3000× *g* for 30 min. The filtrate was analyzed in order to calculate the amount of free GEN still dissolved in the organic phase. GEN loaded into the nanoparticles was calculated by the difference between the amount added and the amount unloaded. Before determining drug loading and entrapment efficiency, GEN adsorption on the Amicon^®^ membrane was evaluated. Solution of GEN (0.5 mg, correspondent to the GEN amount in nanoparticle dispersion) was tested into Amicon^®^ Ultra − 15 (MWCO 30 kDa, Millipore Merck KGaA, Darmstadt, Germany) to evaluate eventual retention of GEN. After centrifugation of the solution at 3000× *g* for 30 min, the concentration of GEN in the filtrate was detected by HPLC following the method described later.

The drug loading (*DL*) (%) was calculated applying the following equation [47]:

$$DL\;\left(\%\right)=\frac{amount\;of\;GEN\;loaded\;in\;nanoparticles}{amount\;of\;GEN\;added}\times \mathrm{theorical}\;DL\;\left(\%\right)$$

The entrapment efficiency was calculated as follows:

$$\%\;EE\;=\frac{\;DL\;}{theorical\;DL}\times 100$$

### 2.7. HPLC Method

To quantify the amount of unloaded GEN in NPAc2 and NPEt1, a modified high performance liquid chromatographic method (HPLC) was employed [48]. Varian ProStar 210 with AutoSampler 410 and a PDA photodiode array detector (Varian Inc Scientific Instruments, Walnut Creek, CA, USA) was used. HPLC analysis was performed using a reverse-phase C-18 column (100 × 4.6 mm; Supelco Ascientis, Millipore Merck KGaA, Darmstadt, Germany) at 25 °C. The binary mobile phase consisted of acetonitrile and 25 mM (350 μL/250 mL) acetic acid solution (50/50, *v*/*v*) which was filtered through 0.45-μm nylon membrane filters (Sartorius, Goettingen, Germany) prior to use. The mobile phase was pumped at a flow rate of 1.2 mL min^−1^. The sample injection volume was 20 µL and all samples were filtered before analysis. The detection of the drug was performed at 260 nm. The GEN content was determined by data extrapolation using a calibration curve obtained with GEN standard solutions. The curve was linear in the range of 0.5–10.0 mg/L (*R*^2^ = 0.9996). The reported values are the mean of three independent analyses.

### 2.8. Evaluation of pH

To confirm a suitable pH for nasal administration (3.5 < pH < 6.4), the pH of the selected nanocarriers was determined at 25 °C with a calibrated pH meter (Eutech Instruments, pH 510, Singapore) [49].

### 2.9. Morphological Analysis

On the basis of results obtained, B-NPEt1 and NPEt1 were selected for further experiments.

The morphology and surface characteristics of nanoparticles were analyzed by scanning electron microscopy, transmission electron microscopy, and atomic force microscopy.

#### 2.9.1. Scanning Electron Microscopy (SEM)

Morphology of nanoparticles compared to the pure drug and polymer were investigated by scanning electron microscopy by means of a FEI Q250 microscope (FEI Company, Dawson Creek, Hillsboro, OR, USA). Thin films of the sample were prepared on a carbon coated copper grid by just dropping a very small amount of the sample on the grid Concerning the nanoparticle dispersions (B-NPEt1 or NPEt1), extra solution was removed using a blotting paper and then the film on the SEM grid were allowed to dry overnight at room temperature. Powders of drug or purified chitosan were directly spread out on the stub before the analysis.

#### 2.9.2. Transmission Electron Microscopy (TEM)

The shape and surface of B-NPEt1 or NPEt1 were investigated by using a Tecnai^®^ G2 F20 Twin TMP (FEI Company, Dawson Creek, Hillsboro, OR, USA). For this, 20 μL of nanoparticle dispersion was placed on the carbon film 200 mesh cooper grid. In order to prevent artefacts, proper drying was performed: small triangles of Whatman paper (Whatman^®^ Cellulose Filter Paper, Sigma-Aldrich, Milan, Italy) were used for a gentle first drying; grids were stored overnight, at room temperature, allowing to sit until the final slow air-drying. Following this drying procedure, samples were stained with 1 M uranyl acetate solution and after drying overnight, the negatively stained structures were examined using the electron microscope. The analysis of TEM data and images was carried out using Digital Micrograph from Gatan and TIA software.

#### 2.9.3. Atomic Force Microscopy (AFM)

The topography of the samples was characterized using an atomic force microscope MFP-3D (Asylum Research, Oxford Instruments company, Santa Barbara, CA, USA). Tips were from Nanosensors model EFM (k¼2N m_1, Ptlr5 coating). Thus, 40 μL of nanoparticle dispersions (B-NPEt1 or NPEt1) were placed on a microscope glass slide and left overnight for water evaporation. After drying, samples were directly analyzed. Images were obtained using Igor Pro6.3.4.1 MFP3D Template software.

### 2.10. X-Ray Analysis

X-ray diffraction (XRD) patterns were obtained using an X-ray diffractometer SMARTLAB diffractometer (Rigaku Europe SE, Neu-Isenburg, Germany) with a rotating anode of Copper working at a power of 40 kV and 100 mA, the diffractometer being also equipped with a graphite monochromator and a scintillation tube.

### 2.11. Ex Vivo Permeation Studies on Nasal Mucosa

Ex-vivo permeability of NPEt1 nanoparticles was assessed using swine nasal mucosa compared to drug suspension in phosphate buffer pH 6.5 containing 50 µg of GEN. Fresh nasal tissue were taken out from the nasal cavities of pigs obtained from the local slaughterhouse (Milia S.r.L, Approval Number CE IT 1856M (Regulation EC 853/2004)). The nasal mucosa was separated from septum. Furthermore, the adhering cartilaginous tissue was carefully removed with without damaging the mucosa. It was used within 1 h after the animals were slaughtered. Tissue samples were inserted in modified Franz diffusion cells [50] with a permeation area of 3.46 cm^2^. Phosphate buffer pH 6.5 was added to the acceptor chamber at 37 °C.

At predetermined time points (0, 15, 30, 60, 90, 120 min) samples were withdrawn from the acceptor compartment and replaced with the same volume of medium. The samples withdrawn were filtered and used for analysis. In the case of NPEt1, the samples were diluted with methanol in order to extract the drug. The amount of permeated drug was determined using HPLC method at 260 nm. Possible analytical interferences were verified. The results were plotted as cumulative drug permeated versus time (*n* = 3 ± SD).

At the end of the ex vivo permeation test, the formulation remaining on the mucosa surface was recovered and analyzed to calculate the amount of un-permeated GEN. The residue was added to methanol and sonicated for 5 min to extract GEN from nanoparticles and to solubilize the dispersed drug. The filtered suspension was analyzed by HPLC. Furthermore, after washing, the mucosa was frozen and then homogenized in an ice bath by an Ultra-Turrax IKA T25 (IKA, Staufen, Germany) in the presence of 5 mL of methanol. Successively the suspension was centrifuged at 3000× *g*. The supernatant was filtered (0.22 µm) and analyzed. The total GEN contents on the permeation surface and inside mucosa were expressed as percentages with respect to the formulation ± SD [51].

### 2.12. In Vitro Cytotoxicity Studies

#### 2.12.1. Cell Culture

PC12 cells, rat pheochromocytoma-derived cell line (ATCCCRL-1721) (passages 12–25) were maintained at 37 °C in atmosphere humidified air 5% CO_2_/95 in 60 mm and cultured in plastic culture plates with Dulbecco’s modified Eagle’s medium supplemented with 10% horse serum, 5% fetal bovine serum, and 1% of penicillin/streptomycin. PC12 cells were treated for 24 h with two series of chitosan nanoparticles (NPAc2 and NPEt1) containing GEN 30 µM, prepared in acetone and ethanol, respectively. The amount of GEN was chosen on the basis of our previous work: it was demonstrated that GEN 30 µM do not induce cytotoxicity; on the contrary, increasing the GEN concentration determines the decreasing PC12 cell vitality [32].

#### 2.12.2. MTT Assay

At the end of the exposure time the cell viability was assessed by MTT (3-(4, 5-dimethyl- thiazol-2-yl)-2, 5, diphenyltetrazoliumbromide) assay. For this, 1 mg/mL of MTT was added for each sample and incubated for 4 h at 37 °C. Only viable cells are able to convert the soluble dye MTT into the insoluble formazan crystals. After the incubation the MTT solution was removed, the cells were washed in phosphate-buffered saline (PBS) and centrifuged, and the pellet was dissolved in 2 mL of isopropanol. The absorbance values were detected by a Bauty Diagnostic Microplate Reader at 578 nm. All experiments were performed in 24-well plates (1 × 10^5^ cells/mL/well) and repeated in triplicate.

#### 2.12.3. Trypan Blue Assay

At the end of the experiments a trypan blue (0.4%) exclusion assay was performed to assess the cell viability evaluating the capability of viable cells to exclude the dye. All experiments were performed in 24-well plates (1 × 10^5^ cells/mL/well) and repeated in triplicate.

#### 2.12.4. Apoptosis Assessment

PC12 cells seeded in 6-well plate at 1.5 × 10^6^ concentration were exposed to B-NPEt1 and NPEt1(containing GEN 30 µm). After 24h of treatment PC12 cells were washed with Annexin V binding buffer. The apoptosis was evaluated using Annexin V-FITC (Sigma Aldrich): in detail, cells were incubated with Annexin V-FITC/PI for 20 min in dark at room temperature (20–25 °C). Cells were analyzed using a FACSCANTO cytometer (BD Bioscience, Franklin Lakes, NJ, USA).

### 2.13. Statistical Analysis

Data are shown in in vitro experiments as mean ± SD. For in vitro and ex vivo studies, at least triplicates were performed. Statistical analysis was performed with Graph-Pad Prism 5.0 software (GraphPad Software, Inc, San Diego, CA, USA). Data were analyzed using unpaired *t*-test and the analysis of variance (one-way ANOVA) followed by a Tukey’s multiple comparison test. The significance level was set at *p* < 0.05, if not stated otherwise.

All experiments on cells are expressed as mean values with 95% confidence intervals and, statistical significance between control and experimental groups was evaluated as significant when two-tailed *p* values were <0.05. All analyses were evaluated by one-way ANOVA analysis of variance test.

## 3. Results and Discussion

### 3.1. Analysis of Particle Size and Polydispersity

The cross-linker SHMP was used to prepare CS nanoparticles through ionic gelation technique, which involves the blending of an alkaline phase in acidic phase. The amino groups of the polymer interact with polyanions to form a gel structure due to inter- and intra-molecular linkages [52].

**Figure 1:**
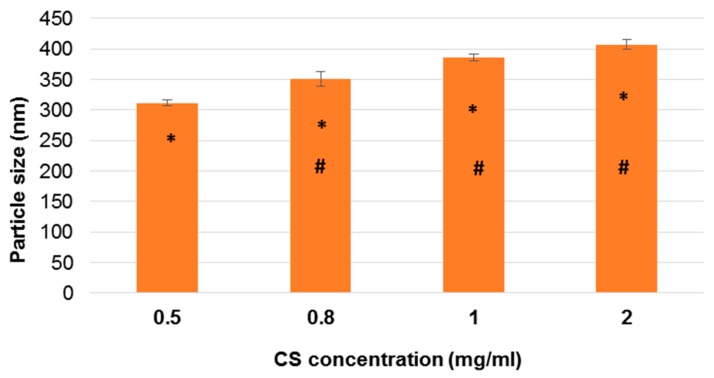
Influence of chitosan (CS) concentration on the particle size. CS:SHMP mass ratio of 4:1 is used. The error bar indicates the standard deviation averaged from three measurements. * *p* < 0.05: 0.5 vs. 0.8, 1, and 2; ^#^ *p* < 0.05: 0.8 vs. 1 and 2. SHMP: sodium hexametaphosphate.

The particle size of CS nanoparticles ranged from 300 nm to 400 nm and was influenced by concentration of polymer: mean diameter increased along with the increase of CS concentration (Figure 1) from 0.5 mg/mL to 2 mg/mL (*p* < 0.05); no statistical differences were observed by using 1 or 2 mg/mL CS concentration *p* > 0.05). Moreover, nanoparticles polydispersity increased with CS concentration, from 0.330 to 0.477, according to Hassani et al. [53]. On the basis of these results, the concentration of chitosan solution was fixed at 0.5 mg/mL.

To investigate the effect of CS and SHMP ratio mass on the dimensional characteristics of nanoparticles and obtain particles with size suitable for nasal application, a new series of nanoparticles were prepared by changing the mass ratio between CS and SHMP.

Results showed particle size values from 694.3 nm to 252.2 nm, meaning that the ratio between CH and SHMP is an essential parameter controlling the dimensional properties of nanoparticles (Table 1).

Due to the formation of particles, the suspension became more turbid by increasing the amount of cross-linker. Nevertheless, the employment of higher concentration of SHMP determined the formation of precipitate. On the contrary, if the amount of SHMP was not present in sufficient amount to complete cross-linking process, the suspension appeared clear as a chitosan solution. In particular, particles decreased in size with increasing mass ratio (Table 1) (*p* < 0.05) with regards to A–D nanoparticles. As D and E particle sizes were not statistically significant (*p* > 0.05), formulation D (with mass ratio 4:1) was chosen as a leader for further studies. Moreover, D showed the lowest PDI value of 0.330 (*p* < 0.05 vs. A, B, C, E), indicating good size distribution properties and therefore an homogeneous dispersion.

On the basis of the diverse dimensional properties of nanoparticles with regard to CS concentration and CS:SHMP mass ratio, GEN-loaded nanoparticles were produced (Table 2).

The amount of GEN loaded was established on the basis of preliminary experiments: briefly, the increase of GEN amount higher than 0.5 mg/mL, when ethanol is used as solvent, leads to the drug precipitation. On the other hand, using acetone it was possible to load up to 2 mg/mL of drug.

The effect of drug loading on particle size and PDI was evaluated and summarized in Table 2. Concerning nanoparticles prepared by dissolving GEN in acetone, the loading of drug determined an increase of mean diameter (*p* < 0.05) (Table 2) corresponding to the amount of GEN loaded.

Nevertheless, GEN-loaded nanoparticles NPAc1 and NPAc2 always showed smaller diameters compared to NPEt1, regardless of the amount of GEN loaded (*p* < 0.05) (Table 2). Also, B-NPAc nanoparticles were smaller than B-NPEt nanoparticles (*p* < 0.05). On the contrary, no significant differences were found between mean diameters of loaded and unloaded nanoparticles when ethanol was used as organic solvent (*p* > 0.05). Size distribution was here expressed as PDI.

PDI values of B-NPAc, NPAc1, and NPAc2 were around 0.2, suggesting that the nanoparticles were in a state of monodispersity distribution [54]. On the contrary, B-NPEt and NPEt1 showed average PDI values around 0.4.

### 3.2. Stability Studies

Stability studies clearly demonstrated that physical stability of nanoparticles depends on temperature, but also on the formulation composition.

Briefly, B-NPAc2 nanoparticles were the most stable regardless of temperature. On the contrary, GEN-loaded nanoparticles were stable at 4 °C in terms of size and size distribution: the mean diameter and PDI did not differ from the initial values after 2 weeks. Nanoparticles obtained by using ethanol as an organic solvent were stable if stored at 25 °C, particularly in terms of particle size.

**Figure 2:**
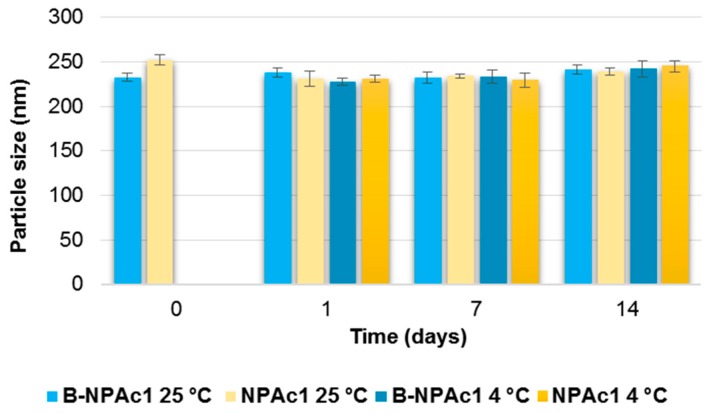
Influence of storage (at 25 °C and 4 °C) on the mean particle size of B-NPAc2 and NPAc2 nanoparticles. *p* < 0.05 NPAc2 at 25 °C: day 0 vs. day 1; day 0 vs. day 7; *p* < 0.05 NPAc2 at 4 °C: day 0 vs. day 1; day 0 vs. day 7.

After stored at room temperature and 4 °C for 2 weeks, the nanoparticles B-NPAc2 were still opalescence and the nanoparticles did not show significant change in particle size (*p* > 0.05) (Figure 2), indicating that unloaded nanoparticles were dimensionally stable regardless of the storage temperature, probably due to appropriate conditions (such as pH and cross-linking density) [55].

With respect to the GEN-loaded nanoparticles included in Figure 2, NPAc2, it can be seen that mean diameter slightly decreases from day 0 to day 1 and day 7 (*p* < 0.05) at both 25 °C and 4 °C, but nanoparticle aggregation occurs after 14 days and particle size values measured after 14 days do not differ from day 0 (*p* > 0.05).

**Figure 3:**
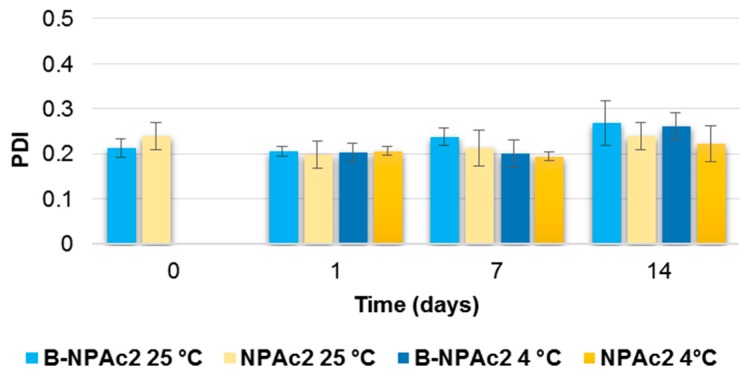
Influence of storage (at 25 °C and 4 °C) on the PDI of B-NPAc2 and NPAc2 nanoparticles. *p* < 0.05 B-NPAc2 at 25 °C: day 1 vs. day 14; *p* < 0.05 B-NPAc2 at 4 °C: day 1 vs. day 14.

Particle size distribution of B-NPAc2 did not change from day 0 to day 1, staying around 0.2; however, the PDI values increased after 14 days (*p* < 0.05) regardless storage temperature. On the contrary, PDI values of GEN-loaded nanoparticles were not modified over time and depending on the temperature (*p* > 0.05) (Figure 3).

**Figure 4:**
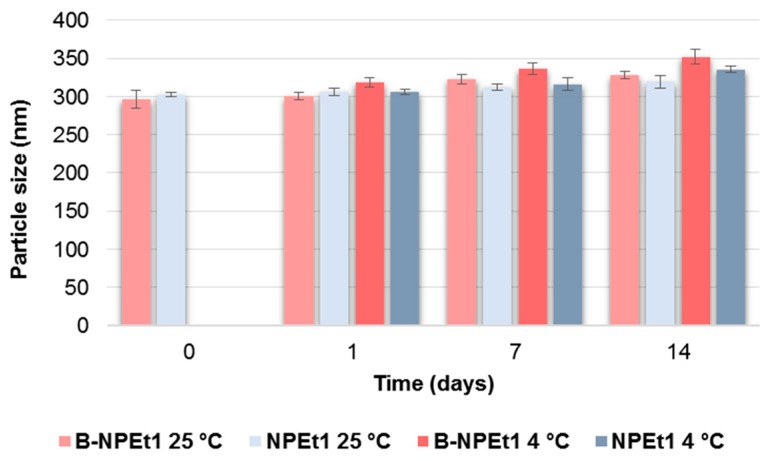
Influence of storage (at 25 °C and 4 °C) on the mean particle size of B-NPEt1 and NPEt1 nanoparticles. *p* < 0.05 B-NPEt1 at 25 °C: day 0 vs. day 7 and day 14, day 1 vs. day 7 and day 14. *p* < 0.05 B-NPEt1 at 4 °C: day 0 vs. day 1, day 7, and day 14, day 1 vs. day 14. *p* < 0.05 NPEt1 at 25 °C: day 0 vs. day 14. *p* < 0.05 B-NPEt1 at 4 °C: day 0 vs. day 7 and day 14, day 1 vs. day 14, day 7 vs. day 14.

B-NPEt1 when stored at 25 °C, showed size stability just for 1 day (*p* > 0.05); then, after 7 days, the mean diameter lightly increased compared to day 0, over 300 nm (*p* < 0.05). Unloaded nanoparticles were less stable by storing at 4 °C, as mean diameter increased over the time reaching 352.1 nm after 14 days (Figure 4).

GEN loading makes nanoparticles more stable at room temperature: the particle size of GEN-loaded formulation NPEt1, increased from 296.7 nm to 335.2 nm but only after 2 weeks (*p* < 0.05). When stored at 4 °C, loaded nanoparticles maintained the size up to day 7; then, small aggregates occurred (*p* < 0.05) (Figure 4).

**Figure 5:**
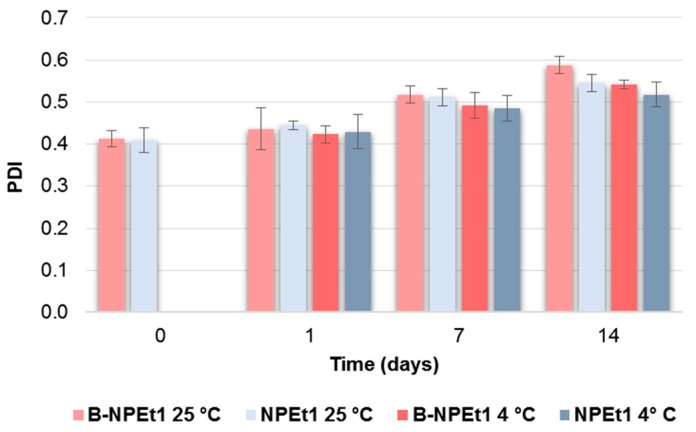
Influence of storage (at 25 °C and 4 °C) on the particle size distribution (PDI) of B-NPEt1 and NPEt1 nanoparticles. *p* < 0.05 B-NPEt1 at 25 °C: day 0 vs. day 7 and day 14, day 1 vs. day 7 and day 14; *p* < 0.05 B-NPEt1 at 4 °C: day 0 vs. day 14, day 1 vs. day 14; *p* < 0.05 NPEt1 at 25 °C: day 0 vs. day 7 and day 14, day 1 vs. day 7 and day 14; *p* < 0.05 NPEt1 at 4 °C: day 0 vs. day 14.

Both unloaded and loaded nanoparticles were more stable and showed homogeneous dispersion by storing at 4 °C, as differences in PDI were not noted before day 14 (*p* < 0.05 day 0 vs. day 14), while PDI increased after day 7 when nanoparticles were stored at 25 °C (*p* < 0.05 day 0 vs. day 7) (Figure 5).

These data allow for the selection of the period of time within which to make further analyses, ensuring a product that dimensionally corresponds to the original.

### 3.3. Determination of the Entrapment Efficiency

In the present study, the entrapment efficiency of GEN was indirectly quantified by measuring GEN content in the supernatant and in the pellet with NPAc2. On the other hand, the amount of unloaded GEN in NPEt1 was measured on the filtrate. In order to evaluate eventual GEN adsorption on the Amicon^®^ membrane, the recovery of GEN in the filtrate was firstly determined; results showed that no loss of GEN occurred.

The percent entrapment efficiency (% EE) of NPAc2 and NPEt1 was found to be 22.9 ± 4.25% and 75.6 ± 7.3%, respectively. Therefore, the EE depends on the organic solvent in which GEN is dissolved. Also, the drug loading was higher when GEN was dissolved in ethanol compared to acetone.

The GEN interactions in nanoparticles can be associated with weak and non-covalent connections, such as hydrogen bond between the hydroxyl groups on the GEN and the free amino groups of the polymer. Ray et al. [56] suggested the drug could be accommodated in the pocket created by physically cross-linking with CS and SHMP.

### 3.4. Evaluation of pH

The pH for NPAc2 and NPEt1 ranged between 4.85 and 5.63, and was therefore within the normal pH range (4.5–6.5) of nasal fluid. The pH of formulations may help reducing the irritation produced upon intranasal administration [57,58].

### 3.5. Morphological Analysis

**Figure 6:**
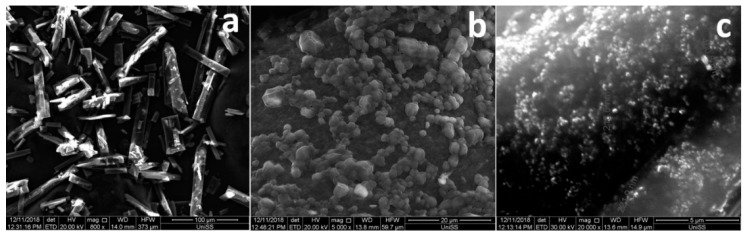
SEM pictures of pure drug (**a**), purified chitosan (**b**), and air-dried nanoparticles (**c**). Magnifications are included in each picture.

**Figure 7:**
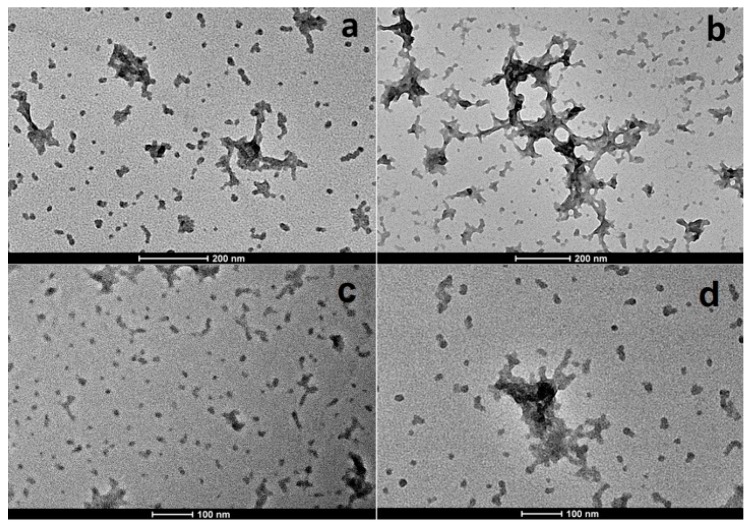
TEM images of loaded (**a**,**c**) and unloaded (**b**,**d**) nanoparticles.

Figure 6 reports SEM pictures of pure drug (a), purified chitosan (b), and air-dried nanoparticles (c). GEN showed crystals with rod-like structure, while purified chitosan was characterised by particles of irregular shape. As concerning loaded and unloaded nanoparticles, they appeared single spherical particles with smooth surface. Nevertheless, TEM images (Figure 7) belied this hypothesis: pictures showed, indeed, the presence of particles in the nano-sized range regardless of drug loading but larger particles in both chitosan nanoparticles can be also observed, probably originating from the aggregation of single small particles as a consequence of the sample-drying procedure. This is according to data in the literature which display air-dried chitosan nanoparticles crosslinked by SHMP [43]. The small particles appeared roundish, while the aggregation process led to irregular necklace-shaped nanoparticles. This behavior seems to be less evident in the sample of loaded nanoparticles compared to unloaded nanoparticles indicating that the drug presence could reduce aggregation process. Moreover, by looking at the size of nanoparticles, it seems there is a discrepancy in the size of nanoparticles between values measured by PCS and TEM micrographs. Particle size observed by TEM seems to be smaller than PCS values; this is in agreement with data in the literature [43]: indeed, the hydrodynamic diameter of nanoparticles is determined in aqueous media, while TEM gives diameter of nanoparticles in dry state.

**Figure 8:**
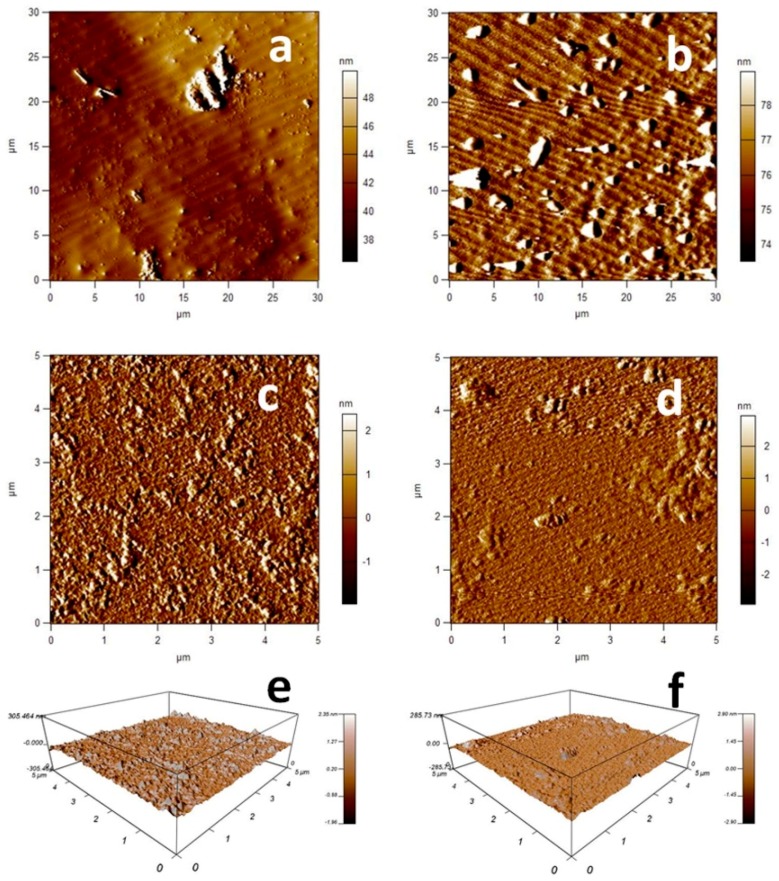
Topographic images of loaded (**a**,**c**) and unloaded (**b**,**d**) nanoparticles. Three-dimensional views of 5 × 5 μm scan of loaded (**e**) and unloaded (**f**) nanoparticles.

A morphological analysis was also carried out by AFM. Figure 8 shows AFM micrographs of loaded and unloaded chitosan nanoparticles. Results confirmed the TEM observations: NPEt1 appeared as small and individual pseudo-spherical particles (Figure 8a) while B-NPEt1 had donut-shaped structure as composed of clusters of nanoparticles (Figure 8b). However, analyzing a smaller area of the samples, it is possible to observe isolated nanoparticles (Figure 8c–f): no morphological and dimensional differences were found between NPEt1 and B-NPEt1.

### 3.6. X-Ray Analysis

**Figure 9:**
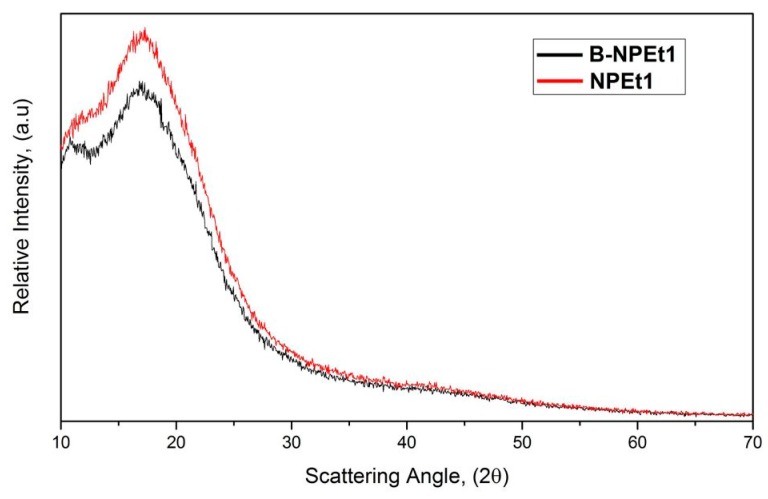
XRD patterns of chitosan nanoparticles prepared with SHMP.

Comparing the XRD pattern chitosan nanoparticles prepared with SHMP (Figure 9), only one broad peak at 2θ = 17.0° was obtained for both loaded and unloaded samples, indicating that formulations had an amorphous structure: it seems that the drug loading into chitosan nanoparticles did not significantly modify the structure of the system even if a slight increase of intensity and change of curve shape occurred, probably determined by an interaction between drug and polymer.

### 3.7. Ex Vivo Permeation Studies on Nasal Mucosa

**Figure 10:**
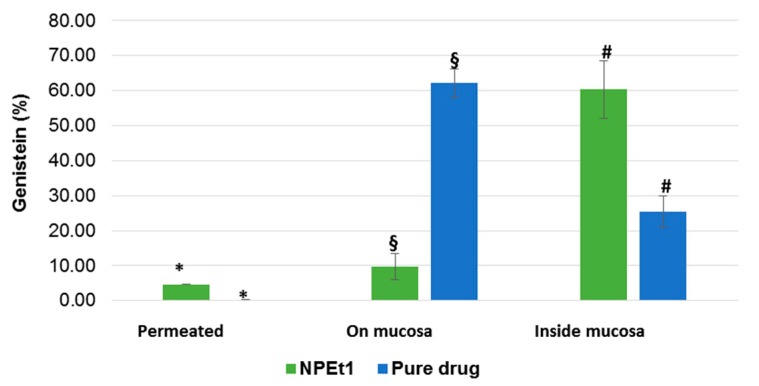
Ex vivo distribution of GEN after the permeation test (120 min) from nanoparticle (NPEt1) and from dispersion in phosphate buffer through the porcine nasal mucosa (*n* = 3). *§# = *p* < 0.05.

The ex vivo permeation test of NPEt1 was carried out and the permeation behavior was compared with that obtained from GEN in dispersion. The Figure 10 shows that free drug in dispersion was mainly recovered on the mucosa surface. Due to the poor hydrophobicity, GEN was not able to cross the cell membranes.

On the contrary, nanoparticles were able to support GEN permeation through the mucosa: even if low amounts of drug loaded into nanoparticles permeated the nasal mucosa (5% of GEN was found in the acceptor medium after 120 min), 60% of the drug was found inside the mucosa. A low percentage (10%) of GEN was still in the residual formulation remained on the tissue.

One of the possible permeation mechanism of the systems can be attributed to the opening of epithelial tight junctions [59] but it cannot be excluded that nanoparticles improve GEN uptake from cells of mucosa as was previously demonstrated by Langasco et al. (2019) [32].

These results demonstrated that over 120 min most of GEN was included into the mucosa, suitable to be released later bypassing the problem of mucociliar clearance and protecting the drug from enzymatic degradation at mucosal surface [36,60].

Therefore, nanoparticles could be employed to increase the permeability and bioavailability of GEN for nose-to-brain administration.

### 3.8. In Vitro Cell Viability Studies

**Figure 11:**
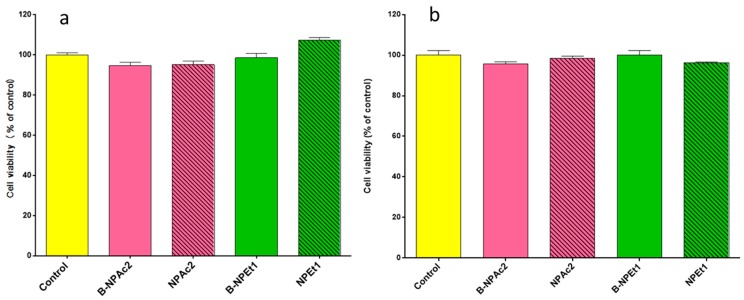
Effects of two different chitosan unloaded (B-NPAc2 and B-NPEt1) and loaded (NPEt1 and NPAc2) nanoparticles prepared in acetone (pink bars) and ethanol (green bars) on PC12 cells viability after 24 h of exposure, evaluated by MTT assay (**a**) and trypan blue assay (**b**).

The two different chitosan formulations GEN-loaded NPEt1 and NPAc2, and unloaded (B-NPAc2 and B-NPEt1) were tested in PC12 cells at 24 h exposure by MTT assay (Figure 11a) and trypan blue assay (Figure 11b). Both MTT and trypan blue analysis showed that all formulations examined did not induce changes in PC12 cells viability (*p* > 0.05). The concentration of GEN was chosen on the basis of the experiments performed in a previous publication [32]. Although nanoparticles pH ranged between 4.85 and 5.63 they did not show decrease in cell viability; this is an important feature as the pH plays a fundamental role in the solubility of chitosan and thus on the stability of nanoparticles. If the pH of nanoparticles should be modified to neutral values, chitosan precipitates and nanoparticles lose their physical stability [61].

**Figure 12:**
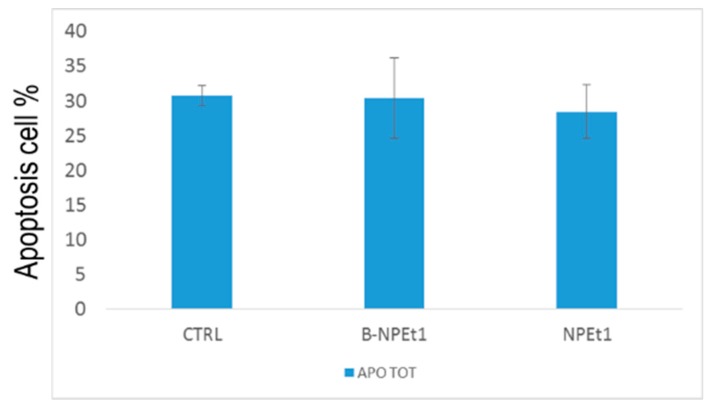
Apoptosis analysis by flow cytometry performed on PC12 cells exposed for 24 h to B-NPEt1 and NPEt1; no significant differences were found between samples (*p* > 0.05 CTRL vs. B-NPEt1 and CTRL vs. NPEt1).

GEN-loaded NPEt1 and unloaded B-NPEt1 nanoparticles were tested in PC12 cells at 24 h to evaluate the effect of nanoparticles on the cell apoptosis. The Figure 12 shows results obtained by the apoptosis analysis where no significance differences in terms of apoptotic events were measured between samples. Moreover, nanoparticles loaded with GEN showed a lower percentage of apoptotic cells than nanoparticles without GEN. These data are in accordance with the values obtained by trypan blue and MTT assay.

## 4. Conclusions

GEN-loaded chitosan nanoparticles were successfully obtained by the ionic gelation method, using SHMP as a valid alternative to most common cross-linkers. Composition of formulation affects the loading efficiency as well as dimensional properties of nanoparticles which are able to promote ex vivo passage of GEN through nasal mucosa and do not show significant changes in terms of cell viability and apoptotic events on PC12 cells. These preliminary but promising results, even if further investigations need to be performed, assess the potential application of these nanocarriers for the delivery of GEN to the brain through nasal route.

## Figures and Tables

**Table 1:** Nanoparticle size properties on the basis of CS:SHMP ratio. The 0.5 mg/mL concentration of chitosan was used.

Formulation	CS:SHMP Mass Ratio	Particle Size (nm ±SD)	PDI (±SD)	Visual Observation
A	2:1	^#ç§^* 694.3 ± 124.1	*^ç#^° 0.625 ± 0.12	Aggregation
B	2.5:1	^#ç§^* 523.2 ± 65.7	^§#^° 0.563 ± 0.08	Opalescence with aggregates
C	3:1	^#ç§^* 385.6 ± 65.7	*^ç#^ 0.476 ± 0.02	Opalescence
D	4:1	^#ç§^* 267.6 ± 23.7	*^§ç#^° 0.330 ± 0.01	Opalescence
E	5:1	^ç§^* 252.2 ± 12.4	*^§#^° 0.421 ± 0.05	Clear

Particle size: * *p* < 0.05: A vs. B, C, D, E; ^§^ *p* < 0.05: B vs. A, C, D, E; ^ç^ *p* < 0.05: C vs. A, B, D, E; ^#^ *p* < 0.05: D vs. A, B, C. Polydispersity index (PDI): * *p* < 0.05: A vs. C, D, E; ^§^ *p* < 0.05: B vs. D, E; ^ç^ *p* < 0.05: C vs. A, D; ^#^ *p* < 0.05: D vs. A, B, C, E; ° *p* < 0.05: E vs. A, B, D.

**Table 2:** Genistein (GEN)-loaded nanoparticles compared to unloaded nanoparticles: composition and dimensional properties (particle size and polydispersity index, PDI).

Formulation	Organic Solvent	GEN (mg)	Particle Size (nm)	PDI
B-NPAc	Acetone	-	*^#^ 232.8 ± 4.3	0.213 ± 0.02
B-NPEt	Ethanol	-	* 286.3 ± 11.2	0.417 ± 0.56
NPAc1	Acetone	1	^ç§#^ 252.4 ± 5.8	0.244 ± 0.03
NPAc2	Acetone	2	^ç§#^ 267.5 ± 9.5	0.229 ± 0.05
NPEt1	Ethanol	0.5	^§^ 296.7 ± 12.3	0.412 ± 0.62

^#^*p* < 0.05 B-NPAc vs. NPAc1 and NPAc2; * *p* < 0.05 B-NPAc vs. B-NPEt; ^§^ *p* < 0.05 NPAc1 vs. NPEt1 and NPAc2 vs. NPEt1; ^Ç^ *p* < 0.05 NPAc1 vs. NPAc2.

## Abbreviations

NDDs	Neurodegenerative diseases
GEN	Genistein
BBB	Blood–brain barrier
H2O2	Hydrogen peroxide
ROS	Reactive oxygen species
PC12 cells	Rat pheochromocytoma-derived cell line
CS	Chitosan
SHMP	Sodium hexametaphosphate
DMEM/F12	Dulbecco’s modified Eagle’s medium
MTT	3-(4,5-dimethyl- thiazol-2-yl)-2, 5, diphenyl tetrazolium bromide
PBS	Phosphate-buffered saline
SD	Standard deviation
B-NPAc, NPAc1, and NPAc2	Unloaded and loaded nanoparticles prepared by adding acetone as organic solvent
B-NPEt and NPEt1	Unloaded and loaded nanoparticles prepared by adding ethanol as organic solvent
PDI	Polydispersity index
PCS	Photo correlation spectroscopy
DL	Drug loading
EE	Entrapment efficiency
HPLC	High performance liquid chromatography
SEM	Scanning electron microscopy
TEM	Transmission electron microscopy
AFM	Atomic force microscopy
